# Trials that turn from retrospectively registered to prospectively registered: a cohort study of “retroactively prospective” clinical trial registration using history data

**DOI:** 10.1186/s13063-024-08029-5

**Published:** 2024-03-14

**Authors:** Martin Holst, Benjamin Gregory Carlisle

**Affiliations:** 1 Berlin Institute of Health at Charité – Universitätsmedizin Berlin, QUEST Center for Responsible Research, Anna-Louisa-Karsch-Str. 2, 10178 Berlin, Germany; 2 Medizinische Hochschule Hannover, Institute for Ethics, History and Philosophy of Medicine, Carl-Neuberg-Str. 1, 30625 Hannover, Germany; 3 Present Address: Department of Equity, Ethics and Policy, McGill University, 2001 McGill College Avenue, Suite 1200, Montreal, QC H3A 1G1 Canada

**Keywords:** Retroactively prospective trial registration, Clinical trials, Trial registries, Start date, Meta-research

## Abstract

**Background:**

Prospective registration of clinical trials is mandated by various regulations. However, clinical trial registries like ClinicalTrials.gov allow registry entries to be updated at any time, and key study elements, including the start date, may change before the first patient is enrolled. If a trial changes its start date after recruiting began, however, it may indicate a reason for concern. This study aimed to measure the rate of “retroactively prospective” trials. This refers to trials that are originally registered retrospectively, with the start date before the registration date, but that retroactively change their start date to be after the registration date, making them appear as if they were prospectively registered.

**Methods:**

We retrieved clinical trial history data for all clinical trials registered on ClinicalTrials.gov with a first registration date in the year 2015 (*N* = 11,908). Using automated analyses, we determined the timepoints of registration in relation to the start date of the trial over time. For retroactively prospective trials and a set of control trials, we manually checked the accompanying publications to determine which start date they report and whether they report changes to the start date.

**Results:**

We found 235 clinical trials to be retroactively prospective, comprising 2.0% of all clinical trials in our sample of 11,908 trials. Among the 113 retroactively prospective clinical trials with an accompanying publication, 12 (10.6%) explicitly stated in the publication that they had been prospectively registered.

**Conclusions:**

Retroactively prospective trial registration happens in one in 50 trials. While these changes to the start date could be mistakes or legitimate edits based on the most up-to-date information, they could also indicate a retrospectively registered trial that has been made to appear as a prospectively registered trial, which would lead to biases unapparent to reviewers. Our results point to the need for more transparent reporting of changes to a trial’s details and have implications for the review and conduct of clinical trials, with our fully automated and freely available tools allowing reviewers or editors to detect these changes.

**Trial registration:**

The preregistered protocol of our study is available via https://osf.io/rvq53. The most recent version of the protocol lists all deviations from the original study plan, including the rationale behind the changes, and additional analyses that were conducted.

**Supplementary Information:**

The online version contains supplementary material available at 10.1186/s13063-024-08029-5.

## Background

Clinical trials provide an important foundation of the medical evidence base [1]. Registration in a public database like ClinicalTrials.gov has become mandated by different regulations: In 2005, the International Committee of Medical Journal Editors (ICMJE) implemented a requirement that all clinical trials be registered before the first patient is enrolled in order to publish in an ICMJE journal [2]. The US Food and Drug Administration Amendments Act of 2007 Sec. 801 requires that certain clinical trials be registered not later than 21 days after the first patient is enrolled [3]. The CONSORT Explanation and Elaboration documentation in 2010 also mentions prospective registration of a clinical trial before the first patient is enrolled [4]. Finally, the Declaration of Helsinki states that “[e]very research study involving human subjects must be registered in a publicly accessible database before recruitment of the first subject” [5]. We will call this practice “prospective registration.”

Prospective registration is important for various reasons: It facilitates a complete and unbiased publication record; informs patients, funders, researchers, and the public of ongoing trials; fulfills ethical obligations; and restricts researcher degrees of freedom in the conduct and analysis of a clinical trial [1, 6–8]. Especially the latter case depends on an unbiased trial record, where all changes to a clinical trial are transparently reported, as mandated by CONSORT [4].

Whether a trial is registered prospectively can be determined by comparing the start date (i.e., the date the first patient is enrolled in the study) to the registration date (i.e., the date the trial registration is submitted to the registry): If the start date is equal to or lies after the registration date, then the trial has been registered prospectively. If the start date lies before the registration date, then the trial has been registered retrospectively. It is important to note, however, that the World Health Organization registry criteria [9] require trial registry platforms to allow updates to the entries, while at the same time maintaining a “publicly accessible audit trail.” This allows investigators to update the start date of their trial, or nearly any other part of the registration record, at any time. Updating the start date does not remove the original trial registry entry version. Instead, on many platforms, it can be found by visiting the publicly accessible historical changes page for the trial in question. This information could so far not be retrieved through most tools provided for downloading clinical trial registry records for larger-scale analysis, as the data of many platforms did not include historical versions. This has changed with the release of the cthist R package [10], which was developed for this project.

Previous research has already dealt with the extent of prospective or retrospective registration of clinical trials [11, 12], finding that between one half and two thirds of the trials are registered retrospectively. Two publications have also dealt with changes to start dates [13, 14], finding that between 7 and 14% have changes to the start date. A change to the start date in itself, however, is not necessarily a reason for concern, with the Final Rule for Clinical Trials Registration and Results Information Submission stating that study start date “means the estimated date on which the clinical trial will be open for recruitment of human subjects, or the actual date on which the first human subject was enrolled” [15]. Instead, it depends on the timing of the change: A change to the start date after it has already passed and the clinical trial has started recruiting would be a possible reason for concern, as one would expect the start date to be fixed by this time. The change might, for example, have been made to turn a retrospectively registered trial into a seemingly prospectively registered trial, to comply with prospective registration policies. In turn, this change would lead to researchers, meta-analysts, editors, reviewers, and the public to be unable to accurately assess potential risks of bias that come with retrospective registration (e.g., undisclosed changes to the primary or secondary outcomes, changes in enrolment). To our knowledge, no study has focused on the timepoint of changes to the start date or on the special case in which clinical trials change their start date after recruiting has started.

**Fig. 1:**
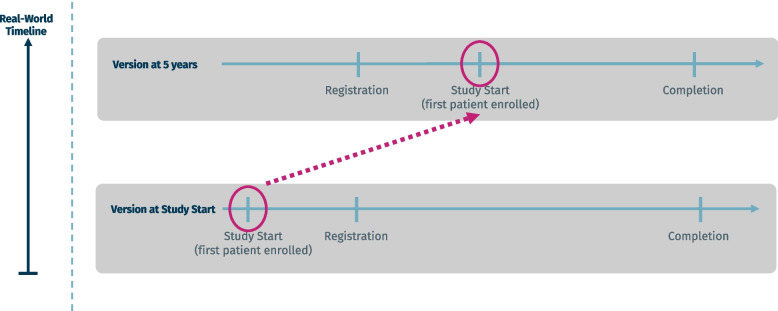
Example for retroactively prospective trial registration. By the time the trial is registered, the trial registry record indicates retrospective registration, but by 5 years, the start date has been pushed forward, indicating prospective registration

In this study, we aimed to determine the frequency of clinical trial records that are originally registered retrospectively (i.e., the first reported start date lies before the registration date), but where the start date is pushed forward in a later registry entry version such that at 5 years after first registration, the trial registry entry indicates prospective registration (i.e., the start of that trial is equal to or dated after the registration date; see Fig. 1). We call these “retroactively prospective” trial registrations. Additionally, we measured further frequencies and proportions and describe whether the numbers differ by phase, sponsorship, or medical field. Furthermore, we estimated the rate of concordance between the registered start date of clinical trials and the start date that is reported in the respective journal publication. Additionally, we aimed to describe how many trials report a change to their start dates, as well as the timepoint of the changes.

## Methods

### Eligibility criteria

In this study, we included studies that met the following criteria: (1) registered on ClinicalTrials.gov (2) between 1 January 2015 and 31 December 2015 (3) as an interventional trial, (4) terminated or completed by 5 years after their first registration date. We focused on one year to ensure an equal follow-up time, and for feasibility considerations regarding computational resources and human rater availability.

### Data sources and sampling

For all trials that met our inclusion criteria, we extracted the complete historical versions using the newly developed cthist R package [10], a tool designed to automatically retrieve historical versions of clinical trial registry records. To ensure equal follow-up, we excluded all historical versions that were posted more than 5 years after registration. We extracted selected data for each trial, most importantly the start date and completion date for each history version, and data about the study phase and the type of lead sponsor. Trials were grouped into phases according to the phase number reported on ClinicalTrials.gov. Trials declared as “Early Phase 1,” “Phase 1,” or “Phase 1 | Phase 2” trials were categorized as Phase 1; all other trials were categorized as Phase 2 and later. Sponsorship was divided into industry-sponsored trials and others.

### Automated analyses

While our sampling was not restricted to any medical field, we determined whether trials examined one of four broad medical fields (cancer, cardiovascular, neurological, pain) by searching for the respective indications on ClinicalTrials.gov and classifying all trials that were found as belonging to that field. We chose these broad fields because they cover a large area of medical research, are broad enough to provide a good sample from each, and have distinct goals and methods. We performed automated analyses to determine the registration status (i.e., retrospective or prospective) of the trials at study start (first registered version after the trial is set to Recruiting, Enrolling by invitation, Active, not recruiting, Completed, or Terminated) and at 5 years (latest history version within the 5-year range). By identifying trials that have an original status as “retrospective registration” and a “prospective registration” status at 5 years, we were able to directly identify trials that constitute retroactively prospective trials, as well as describe changes to the registration status of all other trials, the timepoints of changes, and the time difference between the original start date after launch and the start date at 5 years. Due to changes in policy on ClinicalTrials.gov, some of the study start dates were rounded to the month in the registry entry, while others were exact. We accounted for this by performing separate calculations: If the precision of the start date was only to the month, we used rounded dates for both the start date and the registration date; if the precision of the start date was to the day, we based our calculations on these precise dates.

### Matching trials to publications

All published retroactively prospective trials and a random sample of 200 published trials that were originally prospectively or retrospectively registered, and remained so until 5 years of follow-up, were selected for manual ratings. Using the PubMed application programming interface (API), these trials were automatically matched with journal publications indexed in PubMed that listed the corresponding ClinicalTrials.gov trial number in their “secondary identifier” field or in the text of the title/abstract.

### Manual assessments

For all retroactively prospective trials for which a publication was found as well as our set of control trials with a publication, the downloaded historical versions data were combined with manually extracted data using Numbat Systematic Review Manager [16]. Each corresponding publication was assessed by two of three independent extractors (MH, BGC, SY). We first checked whether the publication was indeed a results publication for the respective trial. Then, we used the full text to extract start dates, end dates, and whether the trial mentioned prospective or retrospective registration. We also extracted whether a change to the start date was mentioned in the publication. Any discrepancies were resolved by discussion.

### Statistical analyses

We used a two-proportion test for equality of proportions with continuity correction to test the hypothesis that clinical trials with retroactively prospective registration would have a lower rate of concordance between the original start date (which would be the first reported start date in retrospectively registered trials and the first reported start date after recruiting began in prospectively registered trials) and the published start date. We used the same test to determine whether there is a difference in the timepoint of changes between retroactively prospective trials and control trials.

### Sample size rationale and calculation

Because most of our analyses can be calculated using data that do not require human curation, we saturated our sampling frame and included all eligible trials that meet our inclusion criteria. For our manually assessed data, we calculated that a sample of at least 200 observations per group was expected to provide 80% power to detect a difference in proportions of 10% and 20% with a significance level of 0.05 in a two-proportion test for equality of proportions with continuity correction.

### Hypotheses

(1) Under the impression of a small pilot search, we hypothesized that there would be a rate of retroactively prospective registrations greater than 5%. (2) We hypothesized that among clinical trials with retroactively prospective registration, there would be a lower concordance between the original start date and the start date reported in the publication, compared to control trials.

### Reporting

Our manuscript adheres to the STROBE Checklist for cross-sectional studies [17].

## Results

**Fig. 2:**
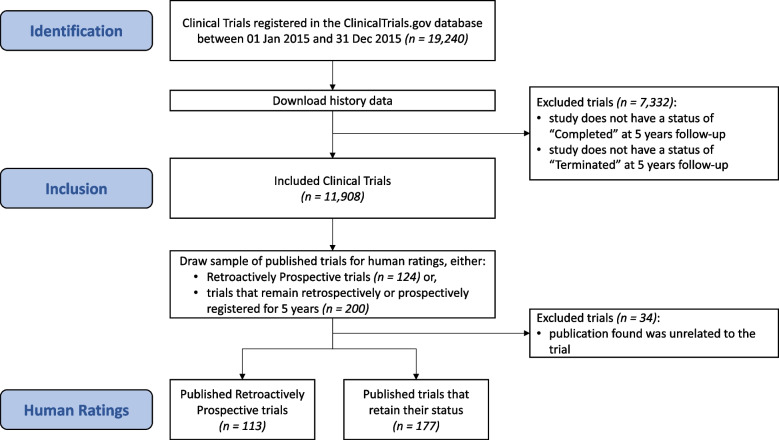
Flowchart of trials

Our flowchart of included trials is shown in Fig. 2. We first downloaded historical versions of the registry data for all included studies on 4 April 2022 (*N* = 11,908, with 147,377 historical versions).

### Number and proportion of retroactively prospective trials

**Fig. 3:**
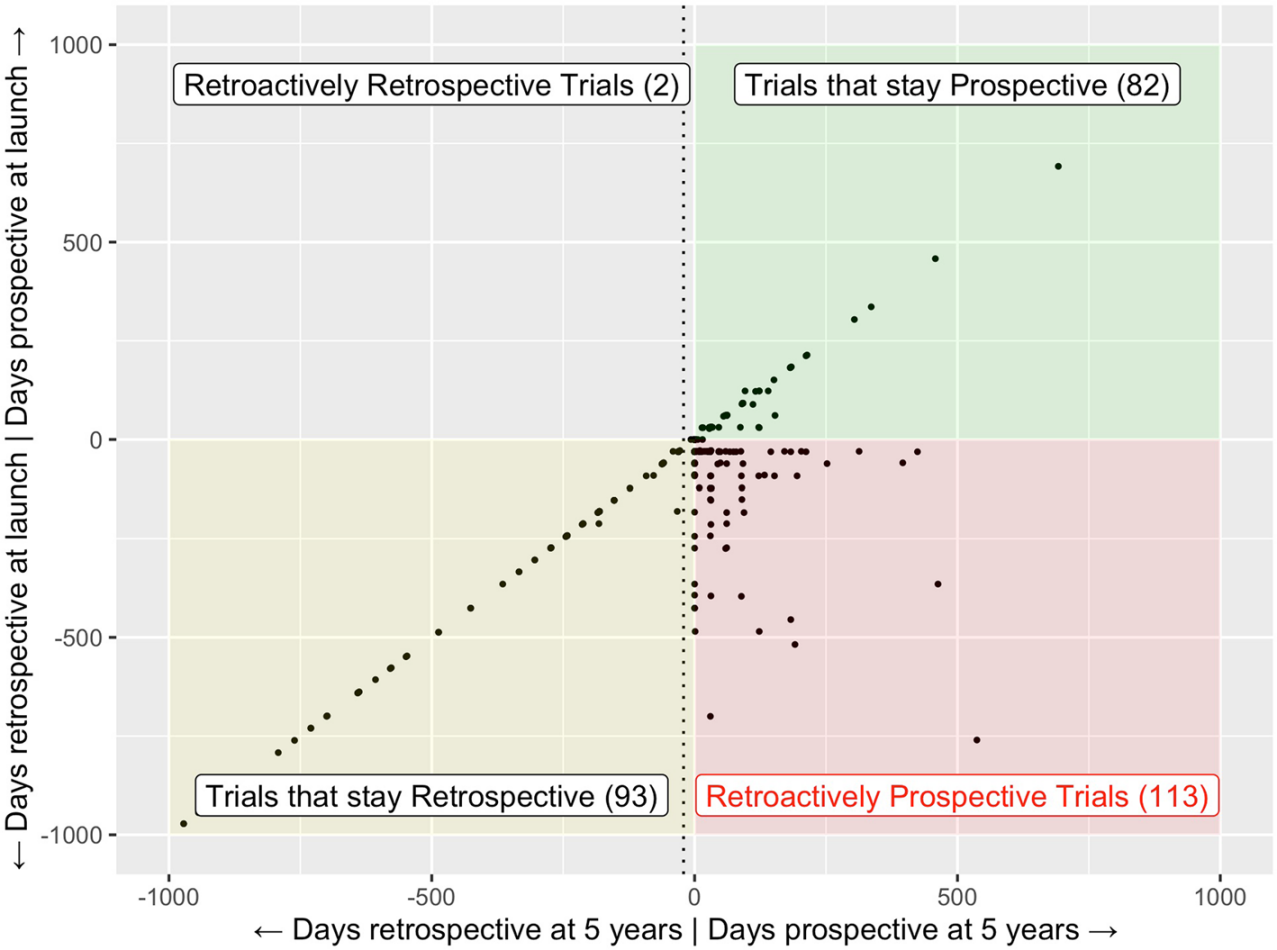
Movements of start dates from launch to 5 years, for 113 published retroactively prospective trials and 177 published controls. Many control trials keep their original start date, and among those that do not, only some of the prospectively registered trials move their start dates around (which would be expected). Among the retroactively prospective trials, however, some get moved forward by quite a bit, with the 5-year start date lying just after the first registration mark. The dotted line represents the 21-day grace period granted by the FDA

We found that 235 out of 11,908 trials (2.0%) had been retroactively prospectively registered, with a start date in the past at the time the study was registered (i.e., retrospective registration), and a start date that lies after the registration date at 5 years later (see also Fig. 3). This did not confirm our first hypothesis. The majority of these retroactively prospective trials switched their start date after the trial had been completed (158 out of 235 trials, which is 67.2% of all retroactively prospective trials). With 50 trials (2.5%), retroactively prospective trial registration was more common in Phase 1 trials.

### Concordance between original registry entries and publications

In a sample of 113 published retroactively prospective and 177 control trials for which a results publication had been found, there was a statistically significant difference in concordance of the original start date with the reported start date in the publication, *χ*^*2*^(1) = 61.346, *p* < 0.001, with 6.0% of the retroactively prospective trials mentioning the original start date in the publication, and 60.9% of the control trials mentioning their original start date in the publication. Thus, our second hypothesis was corroborated.

### Reporting of changes in published trials

In our sample of 113 retroactively prospective trials with an accompanying publication, none of them mentioned a change to the start date in the publication. At the same time, 12 of the 113 published retroactively prospective trials (10.6%) explicitly stated that they had been prospectively registered.

### Timepoints of changes in retroactively prospective trials

To investigate the timepoints of changes, we compared the date of changes of all 235 retroactively prospective trials and all other trials with changes to their start date that were originally registered retrospectively (*n* = 5524). There was a statistically significant difference in proportions of trials that change their start date post-completion, *χ*^*2*^(1) = 717.24, *p* < 0.001, with 67.2% of the retroactively prospective trials changing their start date from retrospective to prospective post-completion, and 9.4% of the control trials having their latest start date change post-completion. Additionally, we calculated at which version number the changes occur: In retroactively prospective trials, the median version number at which the trials are changed from retrospectively registered to prospectively registered was 4, with a median of 5 versions overall (for study results split by phase, sponsorship, and field, as well as additional numbers for all trials investigated, see Supplementary Tables S1 and S2 as well as Supplementary Figure S3).

## Discussion

Our study shows that retroactively prospective trial registration exists, notwithstanding the fact that our primary hypothesis was not corroborated. Almost two percent of trials registered in 2015 had originally been registered retrospectively and changed their start date so that by 5 years after the first registration, they appeared to have been prospectively registered. Thus, when looking at an ostensibly prospectively registered trial from 2015, there is a chance of almost four percent (3.9%) that that trial is in fact a retroactively prospective trial. Many of these trials do not report their original start date in their publication, and none of them report a change to the start date. Twelve of these trials (10.6%) explicitly call their study prospectively registered.

We found that Phase 1 trials had a higher rate of retroactively prospective trials. This is interesting given the fact that the US Food and Drug Administration Amendments Act of 2007 Sec. 801 does not require Phase 1 trials to be prospectively registered. The practice is, however, still mandated by the ICMJE and the Declaration of Helsinki.

Our data show that, in all trials, the start dates fluctuate back and forth (see Supplementary Materials). While it is normal for prospectively registered trials to move their start date around before the actual study start, we found that, at 5 years, some of these trials had become retroactively retrospective, with a start date in the future by the time the study was first registered, but a start date that lies before the registration date at 5 years. We also found start date changes in retrospectively registered trials, which was not according to our expectations, as one would believe that—once the study has started recruiting and the start date is in the past, likely to be known by the trial organizers—there should be little reason to change the start date. These findings certainly underline the difficulty of maintaining an accurate and up-to-date clinical trial registry entry. At this point, we have no evidence to indicate whether these changes were malicious or even intentional. Still, in the aggregate, our analyses suggest that fluctuations in the start dates may be, in some cases, not just mistakes: In retrospectively registered trials, there is a clear bias toward moving the start date forward, and especially after the first registration mark to create a retroactively prospective trial (see Supplementary Figure S3). Also, in most cases, retroactively prospective trials had changed their start date after study completion, and they did so within later history versions. This suggests that, in some trials, the changes happened to comply with editorial or regulatory policies.

There are good reasons to not only register a clinical trial, but to prospectively register it. It fulfills ethical obligations, provides transparent information before the trial is launched (also to funders or regulators), reduces publication bias, and restricts (or makes transparent) researcher degrees of freedom in the selection of hypotheses, outcomes, interventions, or sampling. A trial that is registered prospectively would garner more trust than a trial that was retrospectively registered, as in the latter case, alterations to the study plan might have occurred in the period between study start and first registration, potentially biasing the results. Regardless of whether the change to the start date was a mistake, a correction to an earlier mistake, or a deliberate choice to comply with, for example, journal policies: a trial that appears as a prospectively registered trial while actually possibly being retrospectively registered would not only fail to fulfill the aforementioned obligations, but would also give a false impression regarding the robustness of the study.

Readers, reviewers, or editors cannot determine whether a trial is a retroactively prospective trial from examining the first page of the clinical trial record, or even using the tools provided by ClinicalTrials.gov for mass data download. While the historical data are technically publicly available and the interested reader could visit the ClinicalTrials.gov website and review all historical versions manually, this practice is unfeasible for large sets of clinical trials. This is due to a complex website design, no available API for historical data, and the sheer number of versions to be reviewed (in our sample, there was an average of 6.5 versions per trial, with the maximum number of versions being 180). Thus, even for single trials, tools are needed to help uncover these changes. The cthist R package, which was originally developed for this project, can assist journal editors, peer reviewers, other scientists (including meta-analysts), journalists, or the public in detecting changes to the start dates. It allows these groups to judge more appropriately the bias in a trial and to make appropriate decisions or require disclosures. There is no technical reason why automated solutions that highlight this practice could not be implemented. On the one hand, clinical trial registries could implement solutions to identify and mark these trials, for example, with a badge that says “retrospective registration” or “prospective registration” based on the original start date. Journals, on the other hand, could implement new editorial policies that require editors, peer reviewers, or even specialized personnel to assess the registration status of submitted clinical trials. So far, reviewers check the registry entry of a clinical trial only in a minority of cases [18].

### Limitations

This study is limited in that we only consider trials from ClinicalTrials.gov, which was chosen for its accessibility to historical versions, and because it is by far the largest clinical trial registry. While this allowed us to cover many clinical trials, our findings might not be generalizable to other trials in other registries. Additionally, we limited our search to trials registered in 2015, to ensure an equal follow-up of 5 years and due to constraints in data processing resources and human rater availability. We thus cannot provide any data for other years or for the development over time. The practice of retroactively prospective trial registration might have become less widespread over the years (with more and more journals following ICMJE recommendations [19]), stayed the same, or might even have become more common. In the definition of a study start date, we followed the ClinicalTrials.gov definition, which states that the start date is the “actual date on which the first participant was enrolled in a clinical study.” However, we cannot exclude the possibility that trialists adhere to different, conflicting definitions of the start date (e.g., the start of the funding period). Our method for matching trials to publications depends on journal publications correctly indexing their trial number in PubMed or authors disclosing their trial number in the abstract in accordance with CONSORT [4, 20]. This means that trial publications that were not correctly indexed would not have been included, which might have influenced the results of our sample. Also, we did not allow any “grace period,” counting a trial as retrospective if it had been registered even just 1 day after the study started. US law, for example, allows for a 21-day grace period [21]. Our power calculation resulted in a sample size of 200 per group, based on expected proportions of 10% and 20%. We were, however, unable to reach this sample size, because the number of retroactively prospective trials was lower than expected. However, our effect turned out to be much larger than expected, allowing us to still detect significant results.

### Outlook

Upcoming studies could analyze trends over the years or conduct a survey, asking researchers for the reasons they had to change the start dates or, more broadly, other critical details of the registered study protocol. Studies that have dealt with changes within the registry, or between registry entries and publications [13, 14, 22–26], have to our knowledge not yet surveyed the authors. While we only considered the case of changes to a clinical trial’s start date, similar potential issues exist, as other elements of a trial’s registration can also be changed retroactively. This includes trial outcomes, patient enrolment, eligibility requirements, and other aspects [13, 14, 27]. We chose to analyze registration and start dates because it can be automated, but other registry entry changes could also be similarly analyzed.

## Conclusions

Our study is the first to shine a light on the practice of retroactively prospective registration. This practice could potentially undermine clinical trial transparency and integrity; however, it could be addressed with technical solutions and editorial policies. More than 10% of the retroactively prospective trials with matched publications explicitly indicated that they were prospectively registered and changes to the registered start date were not disclosed in any of these cases. While there are legitimate reasons to change a trial’s registration information, disclosure of such changes will foster confidence and encourage accuracy in clinical trial registrations.

### Supplementary Information

**Additional file 1: Supplementary Table S1.** Numbers and proportions of trials regarding key objectives. **Supplementary Table S2.** Further numbers and proportions of trials.

**Additional file 2:** **Supplementary Figure S3.** Movements of start dates from launch to 5 years, for all trials in our sample. The dotted line represents the 21-day 'grace period' granted by the FDA. To better understand the fluctuations in the start dates, we investigated start date changes in retrospectively registered trials (where one would not expect a change to the start date). Overall, we found 1943 of 5759 retrospectively registered trials (33.7%) to have changed their start date. These trials had a clear bias to the positive, with median difference of 29 days and a mean of 72.4 days (i.e., start dates got pushed forward 72 days on average).

**Additional file 3**.

## Data Availability

The datasets generated and analyzed during the current study, which includes both the publicly available and the manually extracted data, are available in the Open Science Framework repository, https://osf.io/rvq53. The code underlying the analyses of the current study is available via the online repository Codeberg, https://codeberg.org/bgcarlisle/RetroProspectiveCTR.

## Abbreviations

API: Application Programming Interface
CONSORT: Consolidated Standards of Reporting Trials
ICMJE: International Committee of Medical Journal Editors
STROBE: Strengthening the Reporting of Observational Studies in Epidemiology

## References

[1] Zarin DA, Keselman A (2007). Registering a clinical trial in ClinicalTrials.gov. Chest..

[2] De Angelis CD, Drazen JM, Frizelle FA, Haug C, Hoey J, Horton R (2005). Is this clinical trial fully registered? — a statement from the International Committee of Medical Journal Editors. New Engl J Med.

[3] Food and Drug Administration Amendments Act of 2007. 2007.

[4] Moher D, Hopewell S, Schulz KF, Montori V, Gotzsche PC, Devereaux PJ (2010). CONSORT 2010 explanation and elaboration: updated guidelines for reporting parallel group randomised trials. BMJ..

[5] World Medical Association (2013). Declaration of Helsinki: ethical principles for medical research Involving human subjects. JAMA.

[6] Glasziou P, Altman DG, Bossuyt P, Boutron I, Clarke M, Julious S (2014). Reducing waste from incomplete or unusable reports of biomedical research. Lancet.

[7] Ioannidis JPA, Greenland S, Hlatky MA, Khoury MJ, Macleod MR, Moher D (2014). Increasing value and reducing waste in research design, conduct, and analysis. Lancet.

[8] Macleod MR, Michie S, Roberts I, Dirnagl U, Chalmers I, Ioannidis JPA (2014). Biomedical research: increasing value, reducing waste. Lancet.

[9] World Health Organization. WHO Registry Criteria [Internet]. [cited 2023 Oct 3]. Available from: https://www.who.int/clinical-trials-registry-platform/network/registry-criteria

[10] Carlisle BG (2022). Analysis of clinical trial registry entry histories using the novel R package cthist. Naudet F, editor. PLoS ONE..

[11] Haslberger M, Gestrich S, Strech D (2023). Reporting of retrospective registration in clinical trial publications: a cross-sectional study of German trials. BMJ Open.

[12] Harriman SL, Patel J (2016). When are clinical trials registered? An analysis of prospective versus retrospective registration. Trials.

[13] Huić M, Marušić M, Marušić A (2011). Completeness and changes in registered data and reporting bias of randomized controlled trials in ICMJE Journals after trial registration policy. PLoS ONE.

[14] Pranić S, Marušić A (2016). Changes to registration elements and results in a cohort of Clinicaltrials.gov trials were not reflected in published articles. J Clin Epidemiol..

[15] U.S. Health and Human Services Department. Final Rule for Clinical Trials Registration and Results Information Submission (42 CFR Part 11). [Internet]. 81 Federal Register 64982; 2016 [cited 2023 Sep 29]. Available from: https://www.federalregister.gov/documents/2016/09/21/2016-22129/clinical-trials-registration-and-results-information-submission 27658315

[16] Carlisle BG. Numbat Systematic Review Manager [Internet]. The Grey Literature; 2014 [cited 2023 Feb 10]. Available from: https://numbat.bgcarlisle.com

[17] von Elm E, Altman DG, Egger M, Pocock SJ, Gøtzsche PC, Vandenbroucke JP (2007). The Strengthening the Reporting of Observational Studies in Epidemiology (STROBE) statement: guidelines for reporting observational studies. The Lancet.

[18] Mathieu S, Chan AW, Ravaud P (2013). Use of trial register information during the peer review process. PLoS ONE.

[19] International Committee of Medical Journal Editors. Journals stating that they follow the ICMJE Recommendations [Internet]. [cited 2022 Oct 4]. Available from: https://www.icmje.org/journals-following-the-icmje-recommendations/10.12771/emj.2024.e48 PMC12093628 40704003

[20] Schulz KF, Altman DG, Moher D (2010). CONSORT 2010 Statement: updated guidelines for reporting parallel group randomised trials. BMJ..

[21] Tse T, Williams RJ, Zarin DA (2009). Update on registration of clinical trials in ClinicalTrials.gov. Chest..

[22] Goldacre B, Drysdale H, Dale A, Milosevic I, Slade E, Hartley P (2019). COMPare: a prospective cohort study correcting and monitoring 58 misreported trials in real time. Trials.

[23] Stoll M, Mancini A, Hubenschmid L, Dreimüller N, König J, Cuijpers P (2020). Discrepancies from registered protocols and spin occurred frequently in randomized psychotherapy trials—a meta-epidemiologic study. J Clin Epidemiol.

[24] Chan AW, Hróbjartsson A, Haahr MT, Gøtzsche PC, Altman DG (2004). Empirical evidence for selective reporting of outcomes in randomized trials: comparison of protocols to published articles. JAMA.

[25] Huiskens J, Kool BRJ, Bakker J, Bruns ERJ, Jonge SW, Olthof PB (2020). From registration to publication: a study on Dutch academic randomized controlled trials. Res Syn Meth.

[26] Ramagopalan SV, Skingsley AP, Handunnetthi L, Klingel M, Magnus D, Pakpoor J, et al. Prevalence of primary outcome changes in clinical trials registered on ClinicalTrials.gov: a cross-sectional study. F1000Research. 2014;3(77):1–10. 10.12688/f1000research.3784.1 PMC4032105 25075294

[27] Holst M, Haslberger M, Yerunkar S, Strech D, Hemkens LG, Carlisle BG (2023). Frequency of multiple changes to prespecified primary outcomes of clinical trials completed between 2009 and 2017 in German university medical centers: a meta-research study. PLoS Med.

