# Supplementary material for: Trials that turn from retrospectively registered to prospectively registered: a cohort study of “retroactively prospective” clinical trial registration using history data
Source: Trials. 2024 Mar 14;25:189. doi: 10.1186/s13063-024-08029-5 (PMC10938677; doi:10.1186/s13063-024-08029-5)
Supplement: Supplementary file 1 — Additional file 1: Supplementary Table S1. Numbers and proportions of trials regarding key objectives. Supplementary Table S2. Further numbers and proportions of trials. [file 13063_2024_8029_MOESM1_ESM.docx]

***Supplementary Table S1.* Numbers and proportions of trials regarding key objectives.**

| Number  (proportion as fraction of all respective trials) |  |  | Phase | | |  | Sponsor | |  | Medical field ^1^ | | | | |
| --- | --- | --- | --- | --- | --- | --- | --- | --- | --- | --- | --- | --- | --- | --- |
|  |  |  | 1 | 2+ | NA |  | industry | other |  | cancer | cardio | neuro | pain | other |
| Retroactively prospective trials  (%) | 235  (2.0%) |  | 50  (2.4%) | 74  (1.9%) | 111  (1.9%) |  | 63  (1.8%) | 172  (2.1%) |  | 37  (2.1%) | 23  (1.8%) | 39  (2.3%) | 23  (2.7%) | 133  (1.8%) |
| Trials that were always registered prospectively (%) | 5843  (49.1%) |  | 1163  (56.2%) | 2171  (55.7%) | 2509  (42.3%) |  | 2258  (63.2%) | 3585  (43.0%) |  | 972  (56.3%) | 588  (47.0%) | 862  (50.5%) | 391  (45.8%) | 3493  (47.6%) |
| Trials that were always registered retrospectively (%) | 5524  (46.4%) |  | 792  (38.3%) | 1546  (39.7%) | 3186  (53.7%) |  | 1125  (31.5%) | 4399  (52.8%) |  | 666  (38.6%) | 610  (48.8%) | 778  (45.6%) | 428  (50.2%) | 3522  (48.0%) |
| Retroactively retrospective trials ^2^  (%) | 289  (2.4%) |  | 60  (2.9%) | 99  (2.5%) | 130  (2.2%) |  | 122  (3.4%) | 167  (2.0%) |  | 50  (2.9%) | 29  (2.3%) | 29  (1.7%) | 11  (1.3%) | 182  (2.5%) |
| Trials with missing start dates  (%) | 17  (0.1%) |  | 5  (0.2%) | 9  (0.2%) | 3  (0.1%) |  | 7  (0.2%) | 10  (0.1%) |  | 2  (0.1%) | 0  (0.0%) | 0  (0.0%) | 0  (0.0%) | 15  (0.2%) |
| **Total (%)** | **11908**  **(100%)** |  | **2070**  **(100%)** | **3899**  **(100%)** | **5939**  **(100%)** |  | **3575**  **(100%)** | **8333**  **(100%)** |  | **1727**  **(100%)** | **1250**  **(100%)** | **1708**  **(100%)** | **853**  **(100%)** | **7345**  **(100%)** |

^1^ medical fields are not mutually exclusive; a trial can have more than one medical field assigned

^2^ we found 289 trials to be ‘retroactively retrospective’, i.e., they were originally registered before trial launch, but changed their start date such that at 5 years, the trial appeared to be retrospectively registered

***Supplementary Table S2.* Further numbers and proportions of trials.**

| Number  (proportion as indicated) |  |  | Phase | | |  | Sponsor | |  | Medical field ^1^ | | | | |
| --- | --- | --- | --- | --- | --- | --- | --- | --- | --- | --- | --- | --- | --- | --- |
|  |  |  | 1 | 2+ | NA |  | industry | other |  | cancer | cardio | neuro | pain | other |
|  |  |  |  |  |  |  |  |  |  |  |  |  |  |  |
| Retroactively prospective trials ^2^  (% of all prospective trials at 5 years) | 235  (3.9%) |  | 50  (4.1%) | 74  (3.3%) | 111  (4.2%) |  | 63  (2.7%) | 172  (4.6%) |  | 37  (3.7%) | 23  (3.8%) | 39  (4.3%) | 23  (5.6%) | 133  (3.7%) |
| Trials always registered prospectively ^2^ (% of all prospective trials at 5 years) | 5843  (96.1%) |  | 1163  (95.6%) | 2171  (96.7%) | 2509  (95.8%) |  | 2258  (97.3%) | 3585  (95.3%) |  | 972  (96.3%) | 588  (96.2%) | 862  (95.7%) | 391  (94.4%) | 3493  (96.2%) |
| Trials with missing data at start  (% of all prospective trials at 5 years) | 4  (0.1%) |  | 3  (0.3%) | 1  (0.0%) | 0  (0.0%) |  | 0  (0.0%) | 4  (0.1%) |  | 0  (0.0%) | 0  (0.0%) | 0  (0.0%) | 0  (0.0%) | 4  (0.1%) |
| **Total of all prospective trials at 5 years (%)** | **6082**  **(100%)** |  | **1216**  **(100%)** | **2246**  **(100%)** | **2620**  **(100%)** |  | **2321**  **(100%)** | **3761**  **(100%)** |  | **1009**  **(100%)** | **611**  **(100%)** | **901**  **(100%)** | **414**  **(100%)** | **3630**  **(100%)** |
|  |  |  |  |  |  |  |  |  |  |  |  |  |  |  |
| Trials always registered retrospectively ^2^ (% of all retrospective trials at 5 years) | 5524  (94.9%) |  | 792  (92.9%) | 1546  (93.8%) | 3186  (96.0%) |  | 1125  (90.0%) | 4399  (96.3%) |  | 666  (92.9%) | 610  (95.5%) | 778  (96.4%) | 428  (97.5%) | 3522  (95.0%) |
| Retroactively retrospective trials ^2^  (% of all retrospective trials at 5 years) | 289  (5.0%) |  | 60  (7.0%) | 99  (6.0%) | 130  (3.9%) |  | 122  (9.8%) | 167  (3.7%) |  | 50  (7.0%) | 29  (4.5%) | 29  (3.6%) | 11  (2.5%) | 182  (4.9%) |
| Trials with missing data at start  (% of all retrospective trials at 5 years) | 6  (0.1%) |  | 1  (0.1%) | 4  (0.2%) | 1  (0.0%) |  | 3  (0.2%) | 3  (0.1%) |  | 1  (0.1%) | 0  (0.0%) | 0  (0.0%) | 0  (0.0%) | 5  (0.1%) |
| **Total of all retrospective trials at 5 years (%)** | **5819**  **(100%)** |  | **853**  **(100%)** | **1649**  **(100%)** | **3317**  **(100%)** |  | **1250**  **(100%)** | **4569**  **(100%)** |  | **717**  **(100%)** | **639**  **(100%)** | **807**  **(100%)** | **439**  **(100%)** | **3709**  **(100%)** |
|  |  |  |  |  |  |  |  |  |  |  |  |  |  |  |
| Retroactively prospective clinical trials, post-closure (% of all trials) | 158  (1.3%) |  | 25  (1.2%) | 48  (1.2%) | 85  (1.4%) |  | 31  (0.9%) | 127  (1.5%) |  | 19  (1.1%) | 17  (1.4%) | 27  (1.6%) | 19  (2.2%) | 90  (1.2%) |
| Trials registered only after study end  (% of all trials) | 2358  (19.8%) |  | 349  (16.9%) | 675  (17.3%) | 1334  (22.5%) |  | 464  (13.0%) | 1894  (22.7%) |  | 206  (11.9%) | 232  (18.6%) | 273  (16.0%) | 182  (21.3%) | 1639  (22.3%) |

^1^ medical fields are not mutually exclusive; a trial can have more than one medical field assigned

^2^ these numbers can also be found in Supplementary Table S1, but the denominators have changed
