# Supplementary figures and images for: Trials that turn from retrospectively registered to prospectively registered: a cohort study of “retroactively prospective” clinical trial registration using history data
Source: Trials. 2024 Mar 14;25:189. doi: 10.1186/s13063-024-08029-5 (PMC10938677; doi:10.1186/s13063-024-08029-5)

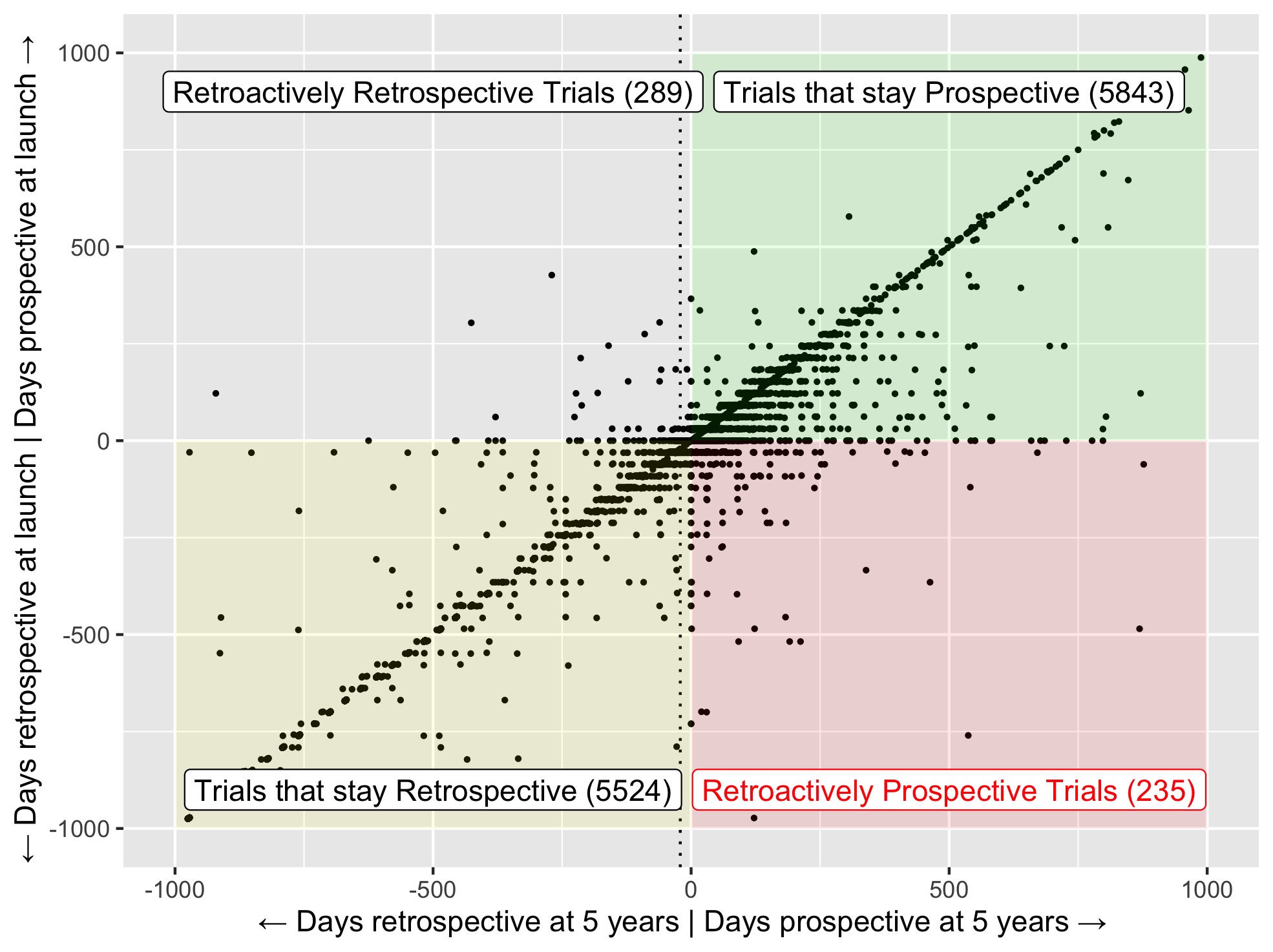

Supplement: Supplementary file 2 — Additional file 2: Supplementary Figure S3. Movements of start dates from launch to 5 years, for all trials in our sample. The dotted line represents the 21-day 'grace period' granted by the FDA. To better understand the fluctuations in the start dates, we investigated start date changes in retrospectively registered trials (where one would not expect a change to the start date). Overall, we found 1943 of 5759 retrospectively registered trials (33.7%) to have changed their start date. These trials had a clear bias to the positive, with median difference of 29 days and a mean of 72.4 days (i.e., start dates got pushed forward 72 days on average). [file 13063_2024_8029_MOESM2_ESM.jpeg]
